# Immune cell profiling in the age of immune checkpoint inhibitors: implications for biomarker discovery and understanding of resistance mechanisms

**DOI:** 10.1007/s00335-018-9757-4

**Published:** 2018-07-02

**Authors:** Su Yin Lim, Helen Rizos

**Affiliations:** 1 Department of Biomedical Sciences, Faculty of Medicine and Health Sciences, Macquarie University, Sydney, NSW Australia; 2 Melanoma Institute Australia, Sydney, NSW Australia

## Abstract

Immunotherapy has changed the landscape of cancer treatment. The introduction of immune checkpoint inhibitors has seen tremendous success in improving overall survival of patients with advanced metastatic cancers and has now become the standard of care for multiple tumor types. However, efficacy of immune checkpoint blockade appears to be limited to immunogenic cancers, and even amongst immune-reactive cancers, response rates are low and variable between patients. Recent data have also demonstrated the rapid emergence of resistance to immune checkpoint inhibitors, with some patients progressing on treatment within one year. Significant research efforts are now directed at identifying predictive biomarkers and mechanisms of resistance to immune checkpoint blockade. These studies are underpinned by comprehensive and detailed profiling of the immune milieu. In this review, we discuss the utility and efficacy of immune cell profiling to uncover biomarkers of response and mechanisms of resistance to immune checkpoint inhibitors.

## Introduction

One of the hallmarks of cancer is the evasion of immune surveillance, arising from the improper monitoring of malignant cells by the immune system due to alterations in oncogenic signaling pathways or changes in the local microenvironment. Cancer cells can downregulate expression of antigens and antigen presentation molecules to hinder immune cell recognition, and conversely, promote expression of immunosuppressive molecules to dampen anti-tumor immune activity. Thus, cancer cells tip the balance towards immune evasion, enabling cancer development and progression (Chen and Mellman 2013, 2017; Vinay et al. 2015; Muenst et al. 2016).

Given that cancers propagate due to dysfunctional immune recognition and activity, several immune-based therapies or immunotherapies that boost immune responses against cancer have been developed. Cytokines such as interferon-alpha2b and interleukin-2 promote cytotoxic T and natural killer (NK) cell activity, and were approved for the treatment of high-risk metastatic melanoma in 1996 and 1998, respectively (Bhatia et al. 2009). The dendritic cell vaccine sipuleucel-T, approved for the treatment of stage IV metastatic prostate cancer, induces cytotoxic T cell responses and led to a 4-month improvement in median overall survival (Kantoff et al. 2010). Other types of vaccines using cancer antigens and adjuvant tumor lysates have been tested in clinical trials with varying efficacy in different cancer types [reviewed in (Finn 2003; Melief et al. 2015; van der Burg et al. 2016)]. Adoptive cell transfer (ACT) (Restifo et al. 2012; Yang and Rosenberg 2016), including chimeric antigen receptor (CAR) T cell therapy (Ramos et al. 2016; Newick et al. 2017), involving the extraction and manipulation of patients’ immune cells, has also improved response rates and survival in certain cancer types.

Amongst the different types of immunotherapies, immune checkpoint inhibitors targeting cytotoxic T lymphocyte-associated antigen 4 (CTLA-4) or programmed death-1/programmed death-ligand 1 (PD-1/PD-L1) signaling have received significant attention in the past 5 years. Under normal conditions, these inhibitory immune checkpoints suppress T cell activity to counteract overactivation of the immune response, and prevent excessive inflammation and tissue damage. However, elevated expression of these inhibitory checkpoints in cancer inhibits anti-tumor T cell function, and immune checkpoint inhibitors are able to mitigate these suppressive effects [reviewed in (Pardoll 2012; Topalian et al. 2015)]. Immune checkpoint inhibitors against CTLA-4, PD-1, and PD-L1 have now been approved by the US Food and Drug Administration (FDA) for the treatment of different cancer types (Table 1).

**Table 1:** Immune checkpoint inhibitors approved by the FDA for the treatment of different cancer types

Drug name	Drug target	Cancer types
Pembrolizumab	PD-1	Advanced (unresectable or metastatic) melanoma
		Advanced non-small cell lung cancer
		Recurrent or metastatic head and neck squamous cell carcinoma
		Locally advanced or metastatic urothelial carcinoma
		Classical Hodgkin’s lymphoma
		Recurrent or metastatic gastric or gastroesophageal junction cancer
		Solid tumor with high microsatellite instability or mismatched repair deficiency
Nivolumab	PD-1	Unresectable or metastatic melanoma
		Adjuvant treatment of melanoma patients with lymph node involvement who have undergone complete resection
		Metastatic non-small cell lung cancer
		Advanced renal cell carcinoma
		Classical Hodgkin’s lymphoma
		Recurrent or metastatic head and neck squamous cell carcinoma
		Locally advanced or metastatic urothelial carcinoma
Avelumab	PD-L1	Metastatic merkel cell carcinoma^a^
		Locally advanced or metastatic urothelial carcinoma^a^
Durvalumab	PD-L1	Locally advanced or metastatic urothelial carcinoma^a^
		Locally advanced non small cell lung cancer^b^
Atezolizumab	PD-L1	Locally advanced or metastatic urothelial carcinoma^a^
		Metastatic non-small cell lung cancer
Ipilimumab	CTLA-4	Unresectable or metastatic melanoma
		Small cell lung cancer

^a^Accelerated approval based on objective response rate and duration of response. Continued approval may depend on further studies and specified disease states

^b^On patients who have not progressed following chemotherapy

Immune checkpoint inhibitors have shown most success in the treatment of advanced metastatic melanoma (McDermott et al. 2014). Treatment with anti-CTLA-4 benefits approximately 20% of patients with advanced melanoma, and a small proportion of these patients remain disease free 10 years after treatment (Schadendorf et al. 2015). Objective response rates are higher with anti-PD-1 treatment (44%) (Robert et al. 2015), and although the combination of anti-CTLA-4 and anti-PD-1 enhances response rate to 58% (Larkin et al. 2015), combination therapy leads to significant treatment-related toxicities (Hodi et al. 2016). Resistance to immune checkpoint inhibitors is an emerging problem, with almost 50% of anti-PD-1-treated patients showing progression within 1 year (Daud et al. 2015).

The limitations of immune checkpoint inhibitors necessitate the identification of biomarkers that can help prognosticate the disease and predict treatment response to improve patient selection and management. Moreover, there is an urgent need to better understand the biological processes underlying the development of resistance to immune checkpoint blockade. In this review, we discuss how profiling of the immune cell contexture can contribute to the discovery of biomarkers and the mechanistic understanding of resistance to immune checkpoint inhibitors.

### Immune cell profiling to uncover biomarkers of response

Cancer biomarkers can provide information on the presence and stage of disease, disease progression and outcomes, and the efficacy and benefits of particular therapies (Kulasingam and Diamandis 2008). Biomarkers can be detected and measured in tissues or blood, and given the importance of immune cell activity in immune checkpoint therapies, there is now a concerted effort to profile and characterize immune cells in tissue and blood biopsies to discover potential biomarkers of response.

### Immune cell counts and proximity

Presence of immune cells can be assessed in tissue biopsies by immunohistochemical (IHC) staining with hematoxylin and eosin, or with antibodies against specific cell surface receptors that are conjugated to different chromogens or fluorescence (Matos et al. 2010). The development of multispectral imaging and detection systems has now enabled multiplex IHC that can detect multiple protein antigens, and differentiate distinct immune cell types in a single tumor sample (Stack et al. 2014; Dixon et al. 2015). This technique offers the added advantage of providing architectural and spatial information about immune cells within the tumor microenvironment. The immune contexture of tumors, encompassing the density, composition, functional state, and organization of immune cells in relation to the tumor, has been shown to provide vital information on disease progression, prognosis, and prediction of treatment response (Fridman et al. 2012, 2017). Indeed, the immunoscore, derived from the density of intratumoral and peritumoral T cells, was shown to be a better prognostic marker in colorectal cancer staging than the tumor, node, and metastasis (TNM) system (Galon et al. 2006, 2014). The immunoscore has also shown prognostic value in other cancer types including breast, prostate, kidney, lung, and melanoma [reviewed in (Galon et al. 2012, 2014; Becht et al. 2016a, b)] and efforts are underway to evaluate its utility in predicting treatment response in other cancer types (Ascierto et al. 2013; Becht et al. 2016a, b). Additionally, although enumeration of T lymphocytes has prognostic significance, identification of specific immune cell subsets is equally important. For example, higher frequencies of CD8 tumor infiltrating T cells are associated with improved clinical outcome and survival (Zhang et al. 2003; Sato et al. 2005), but the presence of T regulatory cells (CD4+ FoxP3+) in tumor tissues is associated with reduced survival and high death hazard in patients with ovarian carcinoma (Curiel et al. 2004).

In melanoma patients, increase in tumor infiltrating lymphocytes (TILs) 3 weeks after treatment with anti-CTLA-4 was associated with clinical activity. Interestingly, high baseline expression of FoxP3, a marker of T regulatory cells, was also associated with clinical activity but expression of CD4, CD8, granzyme B and perforin, markers of effector cytotoxic T cells, did not correlate with response (Hamid et al. 2011). Analysis of CD8 T cell density was also performed on tumor biopsies from 46 melanoma patients receiving anti-PD-1 therapy, at baseline and during treatment. In contrast, intra- and peri-tumoral CD8 T cell density was associated with anti-PD-1 response with AUC ROC (area under the receiver operating characteristic curve) value of > 0.9 (Tumeh et al. 2014). In a separate study, Vilain et al. confirmed that patients responding to anti-PD-1 treatment displayed higher baseline intra-tumoral and peri-tumoral CD8 T cell counts and differences in CD8 T cell counts between responding and non-responding patients were more pronounced in early on treatment biopsies (Vilain et al. 2017). Similarly, Chen et al. also showed significantly higher CD8 T cell density in baseline and particularly, in early on treatment biopsies from melanoma patients responding to sequential anti-CTLA-4 and anti-PD-1 therapy (Chen et al. 2016). Collectively, these studies demonstrate that enumeration and spatiotemporal analysis of CD8 T cells may represent useful biomarkers of response to immune checkpoint blockade.

### Characterisation of immune cell subsets and phenotypes

Although IHC provides structural and spatial information, analysis is restricted to tissue biopsies and this technique alone cannot fully characterize the different immune cell types present in tissues. This is especially limiting for analysis of blood biopsies considering the many different subsets of immune cells circulating in blood. A differential blood count can be used to assess numbers and proportion of white blood cells (typically neutrophils, lymphocytes, monocytes, eosinophils, and basophils), and have been applied to diagnose hematologic malignancies and immune system disorders (Blumenreich 1990). Several recent studies have demonstrated the utility of differential blood count analysis to identify predictive biomarkers of treatment response. For example, low absolute monocyte counts and high absolute eosinophil counts at baseline were associated with a favorable outcome in melanoma patients (*n* = 209) treated with anti-CTLA-4 (Martens et al. 2016). Similarly, high baseline counts of eosinophils and lymphocytes were associated with increased overall survival in melanoma patients (*n* = 616) treated with anti-PD-1 (Weide et al. 2016). Baseline absolute leukocyte and neutrophil counts were also shown to be prognostic in 65 patients with non-small cell lung carcinoma (NSCLC) treated with anti-PD-1 or anti-PD-L1. Specifically, the neutrophil to lymphocyte ratio significantly correlated with survival, and increase in this ratio was prognostic for shorter survival (Mezquita et al. 2017).

There are now mature multiparameter detection systems including flow and mass cytometry that enable the concurrent phenotypic and functional characterization of many different immune cell subsets (Perfetto et al. 2004; Ornatsky et al. 2010). These technologies can be used to investigate immune cell profiles in blood and tissue biopsies, the latter requiring prior processing and disaggregation of tissues into single cells. Flow cytometry uses antibodies that are conjugated to different fluorochromes, and the latest flow cytometer systems allow interrogation of up to 23 parameters using six lasers and 21 florescent detectors (Perfetto et al. 2004). However, flow cytometry is limited by spectral overlap of the fluorescent dyes, whereas mass cytometry overcomes this limitation using antibodies conjugated to heavy metal ions that are subsequently detected by mass spectrometry. Mass cytometry has surpassed the multiplexing capacity of flow cytometry, measuring up to 40 different parameters (Bendall et al. 2012; Spitzer and Nolan 2016), thus allowing the discrimination of different immune cell subsets and phenotypic characterization of their activation, proliferation, and tolerance states. For example, expression of immune exhaustion markers (e.g., PD-1, LAG3, TIM3) may indicate T cell anergy (Baitsch et al. 2011), and analysis of transcription factors and phosphorylated proteins following cell permeabilisation can provide additional information on signaling pathways in immune cells. These cytometry platforms can also incorporate identification of peptide-MHC multimers to characterize T cell reactivity, and cell cycle analysis to determine cell proliferation and viability [reviewed in (Krutzik et al. 2004; Covey and Cesano 2010; Andersen et al. 2012; Bjornson et al. 2013; Newell 2013)]. Advanced analytical platforms such as viSNE (Amir el et al. 2013) and SPADE (Qiu et al. 2011) are now available to allow phenotypic visualization of the high-dimensional single-cell data generated from flow and mass cytometry.

**Fig. 1:**
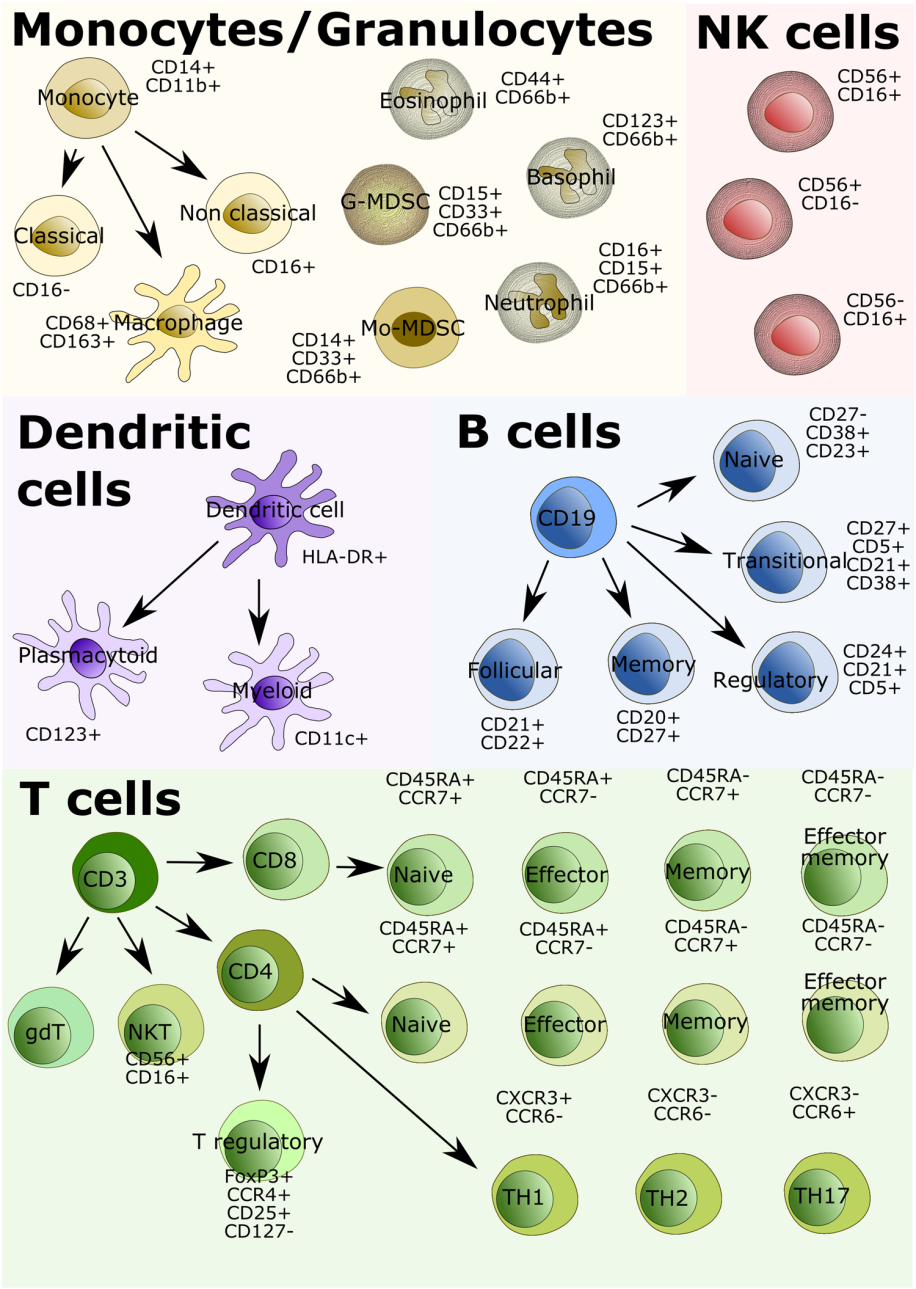
Proposed flow and mass cytometry marker panels for the identification and characterization of major immune cell subsets, including monocytes, granulocytes, natural killer, dendritic, B and T cells. G-MDSC denotes granulocytic myeloid-derived suppressor cell while Mo-MDSC denotes monocytic myeloid-derived suppressor cell

Standardized panels for whole blood immune cell phenotyping using flow or mass cytometry have been proposed and used for the diagnosis of blood-based cancers such as leukemia (Maecker et al. 2012; van Dongen et al. 2012). These panels can be similarly applied and modified to evaluate major populations of distinct immune cell subsets in blood and tissues of patients treated with immune checkpoint inhibitors to uncover potential cell-based biomarkers (Fig. 1). For instance, flow cytometric analysis of peripheral blood mononuclear cells (PBMCs) from 36 metastatic melanoma patients revealed increased frequency of ICOS + CD4 T cells following treatment with anti-CTLA-4, suggesting that this effector T cell population may function as a pharmacodynamic marker of anti-CTLA-4 therapy (Ng Tang et al. 2013). Comparison of PBMC profiles from melanoma patients treated with anti-PD-1 or anti-CTLA-4 showed distinct frequencies of immune cell subsets that may serve as predictive biomarkers. For example, CD4 and CD8 memory T cells were enriched in patients who responded to anti-CTLA-4 therapy whereas increased CD69 + NK cell counts correlated with clinical response to anti-PD-1 therapy (Subrahmanyam et al. 2018). Further, flow cytometric profiling of PBMCs from 29 stage IV melanoma patients before and after treatment with anti-PD-1 identified a reinvigorated exhausted Ki67 + CD8 T cell population. A ratio greater than 1.94 of this CD8 T cell subset to tumor burden significantly correlated with better objective response, progression free survival, and overall survival (Huang et al. 2017). Similarly, mass cytometric analysis of PBMCs from 20 melanoma patients at baseline and 12 weeks after anti-PD-1 therapy revealed frequency of a monocytic population (CD14^+^ CD16^−^ HLA-DR^hi^) to be a strong predictor of progression free and overall survival (Krieg et al. 2018).

Flow or mass cytometric profiling can also be performed on tissue biopsies to expose potential biomarkers. Daud et al. showed significant correlation between CD8 T cells expressing high levels of CTLA-4 and PD-1 with clinical response and progression free survival in freshly isolated metastatic melanoma samples from 40 patients prior to treatment with anti-PD-1, suggesting that relative abundance of this T cell population may predict treatment response (Daud et al. 2016). It is worth mentioning that there may be differences in immune cell counts in peripheral blood compared to the tissue microenvironment, and these differences may reflect immune cell egress from circulation into the tumor tissue.

### Transcriptomic biomarkers of immune cells

Transcriptomic profiling of immune cells in fresh frozen and paraffin embedded tissues using RNA sequencing, microarrays, or the NanoString nCounter platform has provided comprehensive gene expression data on immune cell types and abundance, molecular mechanisms of immune cell activation and behavior, and the inflammatory state of the tumor microenvironment.

Expressions of a specific gene or gene sets similar to the gene panels offered by NanoString (Table 2) are used to differentiate immune cell subsets, including lymphocytic T and B cells, natural killer and dendritic cells, and myeloid cells comprising of neutrophils, macrophages, and eosinophils. However, transcriptome data are derived from heterogeneous cell types in the tumor including malignant, stromal, and immune cells. Several computational algorithms have been designed to deconvolute gene expression data from complex tissue biopsies to estimate immune cell frequencies [reviewed in (Finotello and Trajanoski 2018)]. Programs such as TIMER (Li et al. 2016) and CIBERSORT (Newman et al. 2015) are publicly available and provide estimations of the composition and degree of immune cell infiltration.

**Table 2:** NanoString gene panels used to define specific immune cell subsets in tissue biopsies

Immune cell type	NanoString gene panel
T cells	CD2, CD3E, CD3G, CD6
Helper T cells	ANP32B, BATF, NUP107, CD28, ICOS
TH1	CD38, CSF2, IFNG, IL12RB2, LTA, CTLA4, TXB21, STAT4
TH2	CXCR6, GATA3, IL26, LAIR2, PMCH, SMAD2, STAT6
TH17	IL17A, IL17RA, RORC
Follicular helper T cells	CXCL13, MAF, PDCD1, BCL6
Memory T cells	
Central memory T cells	ATM, DOCK9, NEFL, REPS1, USP9Y
Effector memory T cells	AKT3, CCR2, EWSR1, LTK, NFATC4
Regulatory T cells	FOXP3
Cytotoxic CD8 T cells	CD8A, CD8B, FLT3LG, GZMM, PRF1
Gamma delta T cellls	CD160, FEZ1, TARP
B cells	BLK, CD19, CR2, HLA-DOB, MS4A1, TNFRSF17
Natural killer cells	BCL2, FUT5, NCR1, ZNF205
CD56 high	FOXJ1, MPPED1, PLA2G6, RRAD
CD56 low	GTF3C1, GZMB, IL2IR
Dendritic cells	
Myeloid dendritic cells	CCL13, CCL17, CCL22, CD209, HSD11B1
Immature dendritic cells	CD1A, CD1B, CD1E, F13A1, SYT17
Activated dendritic cells	CCL1, EBI3, IDO1, LAMP3, OAS3
Plasmacytoid dendritic cells	IL3RA
Myeloid cells	
Macrophages	APOE, CCL7, CD68, CHIT1, CXCL5, MARCO, MSR1
Mast cells	CMA1, CTSG, KIT, MS4A2, PRG2, TPSAB1
Neutrophils	CSF3R, FPR2, MME
Eosinophils	CCR3, IL5RA, PTGDR2, SMPD3, THBS1

In addition to immune cell abundance, immune phenotypes can also be discerned based on expression of specific cytokines and mediators (Becker et al. 2015), and the Immunological Genome Project recently aimed to decipher the signaling networks of 249 immune cell types to uncover molecular pathways involved in immune response and interaction (Heng et al. 2008). Combination of genes has also been used to infer immune activity. For example, the geometric mean expression of *PRF1* and *GZMA* transcripts was shown to correlate with cytolytic activity of immune infiltrates, and this cytolytic (CYT) score associated with survival benefit in a range of cancer types (Rooney et al. 2015). Several immune cell signatures that reflect immune differentiation, activation, and signaling have also been proposed (Shaffer et al. 2001; Critchley-Thorne et al. 2011; Godec et al. 2016). Expression of these immune response gene sets, which include antigen presentation molecules (i.e., major histocompatibility complex molecules), interferon signaling effectors, T cell activation, adaptive and innate immunity genes was shown to correlate with prolonged survival in metastatic melanoma patients (Mandruzzato et al. 2006; Bogunovic et al. 2009), relapse free survival in patients with small cell lung cancer (Roepman et al. 2009), and extended time to relapse and recurrence in colon cancer patients (Galon et al. 2006).

In patients treated with immune checkpoint inhibitors, gene expression profiles and signatures reflective of an active immune microenvironment have been shown to correlate with clinical activity [reviewed in (Gajewski et al. 2010; Ulloa-Montoya et al. 2013)], and may serve as biomarkers of treatment response. For example, transcriptome analysis of tumor biopsies from 40 melanoma patients before treatment with anti-CTLA-4 indicated higher expression of the CYT score, CTLA-4, PD-1, PD-L1, and PD-L2 in patients with clinical benefit (Van Allen et al. 2015). Similarly, baseline expression of immune-associated genes including T cell surface markers (CD8, CD3, CD38), cytokines involved in T cell recruitment (CXCL9 and CXCL10), immune receptors (CXCR6 and CCR5), and TNF signaling components correlated with response to anti-CTLA-4 therapy and overall survival, and these associations were more pronounced in early on treatment biopsies (3 weeks after treatment initiation) (Ji et al. 2012). Transcriptomic profiling of longitudinal tumor biopsies allows investigation into the dynamics of immune response during treatment, and in a cohort of melanoma patients treated with anti-PD-1 (*n* = 45), comparison of baseline to on treatment biopsies revealed increased induction of immune-related genes in patients with clinical benefit (Riaz et al. 2017). Similarly, Chen et al. showed significant upregulation of genes associated with IFNγ signaling, antigen presentation, and T cell effector function in on treatment biopsies from 54 melanoma patients treated with sequential anti-CTLA-4 and anti-PD-1 therapies (Chen et al. 2016).

The IFNγ signaling pathway regulates numerous aspects of the immune response, including expression of immune checkpoint proteins, antigen processing and presentation molecules, and various chemokines involved in immune cell recruitment. Several components of the IFNγ signaling pathway including STAT1, STAT2, STAT3, and IRF1 were upregulated in anti-PD-1 responding tumors (Garcia-Diaz et al. 2017), and loss of function mutations in JAK1/JAK2 have been implicated to confer resistance to anti-PD-1 therapy (Zaretsky et al. 2016; Shin et al. 2017). As such, expression of IFNγ pathway signatures may represent surrogate markers of immune activity and response to immune checkpoint therapy. Indeed, high IFNγ expression was shown to be associated with longer progression free survival in non-small cell lung cancer (NSCLC) and melanoma patients treated with anti-PD-1 (Karachaliou et al. 2018). In keeping with this, expression of a 10-gene IFNγ signature was initially interrogated in baseline tumor biopsies from 19 melanoma patients treated with anti-PD-1. Expression of a 28-gene expanded immune signature that incorporated the 10 IFNγ genes as well as T cell and antigen presentation molecules was further validated in a separate cohort of 62 melanoma patients. Both these signatures showed significant association with overall response rate and progression free survival (Ribas et al. 2016), and when examined in baseline tumor samples from head and neck squamous cell carcinoma (HNSCC; *n* = 43) and gastric cancer (*n* = 33) patients treated with anti-PD-1, these signatures also correlated with overall response rate and progression free survival (Ayers et al. 2017). A similar T effector IFNγ gene signature was investigated in NSCLC patients treated with anti-PD-L1 (*n* = 142), and high expression of the signature at baseline correlated with improved overall survival (Fehrenbacher et al. 2016). However, it is important to mention that expression of IFNγ-associated signatures do not always associate with clinical benefit. For example, the T effector IFNγ signature showed no association with clinical benefit in 70 renal cell carcinoma patients treated with anti-PD-L1 (McDermott et al. 2016).

Recently, an IPRES signature, comprised of genes involved in mesenchymal transition, extracellular matrix remodeling, angiogenesis, and wound healing, was shown to be enriched in baseline biopsies of melanoma patients with innate resistance to anti-PD-1 therapy (Hugo et al. 2017). Although co-enrichment of this signature was identified across other cancer types (Hugo et al. 2017), its expression was not associated with innate resistance to anti-CTLA-4 therapy, and thus far, correlation of the IPRES signature with resistance has not been found in other cohorts of patients treated with immune checkpoint blockade (Van Allen et al. 2015; Chen et al. 2016; Riaz et al. 2017).

Transcription profiling can also be performed on homogenous cells instead of admix tissue biopsies. Immune cells can be first isolated from tumor tissues using laser capture microdissection, or tumor dissociation followed by single cell sorting and immune cell enrichment before RNA sequencing. A recent study by Tirosh et al. conducted single-cell RNA sequencing on tumor, immune, and stromal cells isolated from melanoma tumors and reported distinct T cell functional states from the isolated immune cells (Tirosh et al. 2016). Sequencing of the T cell receptor can also be performed to identify T cell repertoire and clonal expansion (Postow et al. 2015; Riaz et al. 2017). Riaz et al. identified temporal changes in the intratumoral TCR repertoire indicative of T cell clonal expansion during anti-PD-1 treatment. In melanoma patients who have progressed on anti-CTLA-4 therapy and subsequently treated with anti-PD-1 (*n* = 35), an increase in the number of unique CDR3 sequences (richness) was associated with clinical benefit (Riaz et al. 2017). Additionally, Postow et al. showed that increased TCR repertoire and T cell diversity in peripheral blood of 12 melanoma patients before anti-CTLA-4 therapy was associated with clinical benefit (Postow et al. 2015).

### Immune cell profiling to elucidate resistance mechanisms

Resistance to immune checkpoint inhibitors may be innate, whereby patients do not respond to therapy at first instance, or acquired, in which patients responded initially but subsequently progress. Innate and acquired resistance mechanisms can be tumor intrinsic, involving changes in the cancer cells, or tumor extrinsic, attributed to alterations in the tumor micro-environment (Sharma et al. 2017). Tumor intrinsic mechanisms include downregulation or loss of antigens and antigen presentation molecules (Zaretsky et al. 2016), disruption in the IFNγ signaling pathway (Zaretsky et al. 2016; Garcia-Diaz 2017) and T cell exclusion (Liu et al. 2013; Peng et al. 2016). Tumor extrinsic mechanisms involve inhibition of innate and adaptive immune cell function and infiltration, and enrichment of immunosuppressive cells or molecules [reviewed in (Sharma et al. 2017; Jenkins et al. 2018)].

**Fig. 2:**
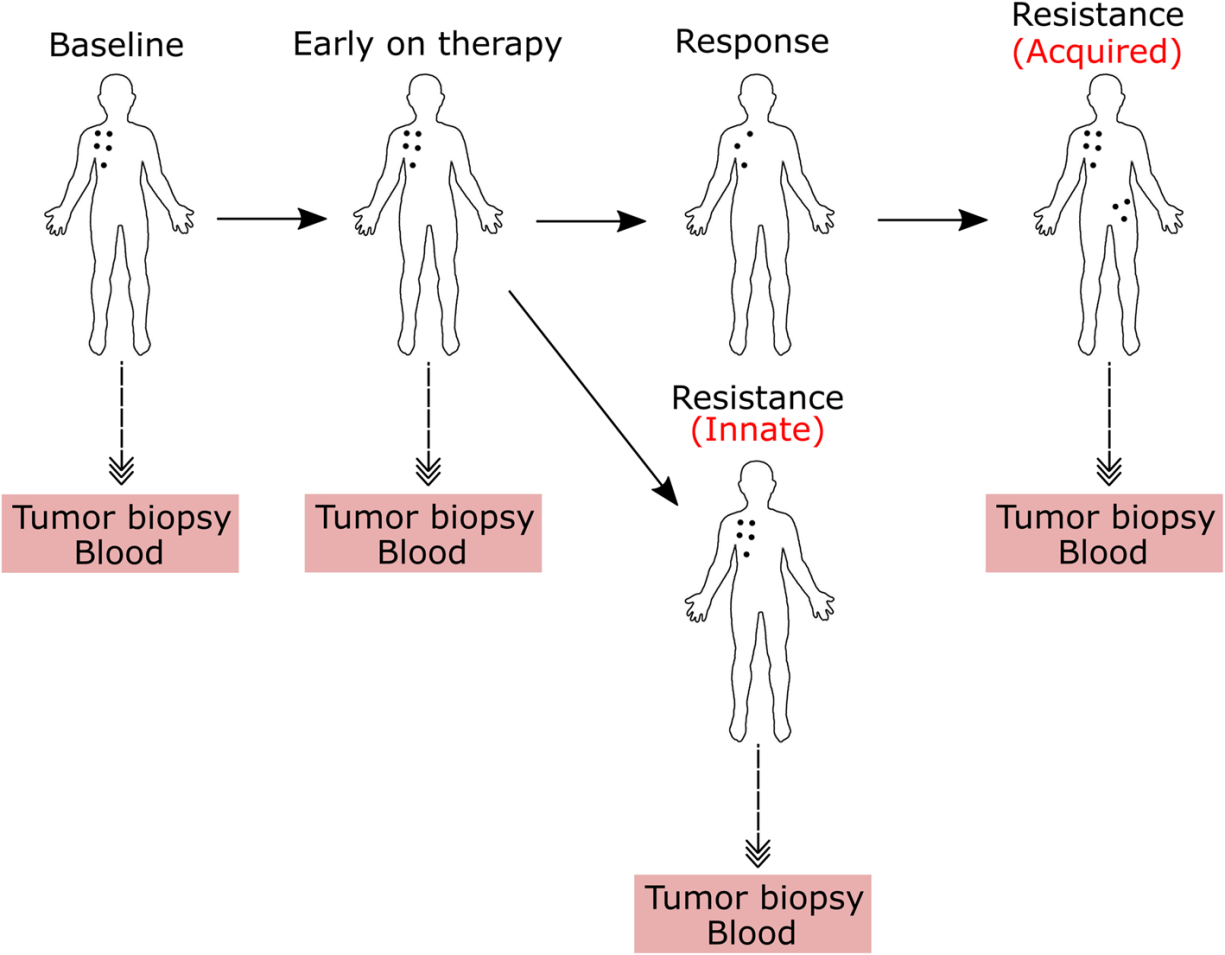
Tissue and blood biopsies can be obtained before therapy (baseline), early during therapy and on progression to study innate and acquired resistance mechanism of immune checkpoint blockade

Immune cell profiling can reveal how tumor intrinsic and extrinsic mechanisms hinder anti-tumor responses and contribute to immune checkpoint therapy resistance. A valuable approach to uncover resistance mechanisms involves immune profiling of longitudinal samples. Comparison of the immune contexture in tissues and blood before and during immune checkpoint therapy can provide information on the dynamics of the immune response and expose defective or abnormal immune activity prior to and throughout the course of therapy (Fig. 2).

### Defective IFNγ responses

The IFNγ signaling pathway acts as a double-edged sword, with critical roles in mediating the degree and extent of anti-tumor immune response, and by extension, the efficacy of immune checkpoint therapy. For example, IFNγ regulates CD8 T cell expansion but can also induce adaptive immune resistance mechanisms. Alterations in IFNγ pathway components that cause dysfunctional or defective signaling are often associated with resistance to immune checkpoint inhibitors. For instance, loss-of-function mutations in the *PBRM1* gene, which encodes a subunit of the PBAF SWI/SNF chromatin remodeling complex, was enriched in tumors of metastatic renal cell carcinoma patients responding to anti-PD-1 therapy. PBRM1/PBAF deficient tumors showed enhanced expression of IL-6 JAK-STAT3 signaling, suggesting that inhibition of this complex may potentiate IFNγ-dependent immune stimulation (Miao et al. 2018). In line with this, Pan et al. identified inactivation of *PBRM1, ARID2*, and *BRD7*, components of the PBAF SWI/SNF complex, to render tumor cells sensitive to IFNγ and T cell-mediated killing. Flow cytometric analysis of *PBRM1*-deficient tumors showed increased infiltration of CD4 T and CD8 T cells, and single-cell RNA sequencing analysis of the immune cells indicated increased expression of immune activation pathways. Importantly, *PBRM1* deletion caused treatment resistant tumors to become responsive to immune checkpoint inhibitors (Pan et al. 2018).

Deletion of a protein tyrosine phosphatase PTPN2 has also been shown to sensitize tumors to immune checkpoint inhibitors, through enhanced IFNγ signaling (Manguso et al. 2017). Similarly, loss of function mutations in *APLNR* gene was identified in non-responding tumor lesions of metastatic melanoma and lung cancer patients treated with anti-PD-1 or anti-CTLA-4 therapy. APLNR was shown to bind JAK1 and induce IFNγ signaling, and its deletion reduced the efficacy of immune checkpoint blockade (Patel et al. 2017). Collectively, these studies utilize immune profiling to demonstrate how dysregulated IFNγ signaling shapes the immune microenvironment and contributes to resistance to immune checkpoint therapy.

### T cell exclusion

Alteration in certain oncogenic signaling pathways has been shown to contribute to T cell exclusion, and hence, proposed as a tumor intrinsic resistance mechanism. PTEN loss in cancer cells was shown to reduce accumulation of adoptively transferred T cells and suppress anti-tumor T cell killing in a mouse model. Loss of PTEN, which leads to activation of the PI3K signaling pathway, was also associated with significantly reduced CD8 T cell infiltration in tumor biopsies of metastatic melanoma patients (Peng et al. 2016). The B-catenin/WNT signaling pathway in cancer cells was similarly implicated in T cell exclusion. Activation of WNT signaling suppressed CCL4 expression, leading to reduced recruitment of CD103+ dendritic cells (Spranger et al. 2015), an antigen-presenting immune cell subset essential for effector T cell trafficking into the tumor microenvironment (Spranger et al. 2017).

### Inhibition of immune cell activity

Immune cell profiling using flow or mass cytometry provides comprehensive characterization of immune cell phenotype, function, and activity, and can elucidate response and resistance mechanisms of immune checkpoint therapy. For instance, mass cytometry profiling of tumor-infiltrating T cells revealed differences in the T cell phenotypes of patients treated with anti-PD-1 compared to anti-CTLA-4. Treatment with anti-PD-1 appeared to invigorate exhausted CD8 T cells while anti-CTLA-4 treatment induced expression of Th1 CD4 T cells that recruit and activate other effector cytotoxic cells, indicating distinct response mechanisms in the two checkpoint inhibitors (Wei et al. 2017).

On the other hand, resistance to immune checkpoint blockade appears to depend on the balance between T cell activity and its inhibition. Profiling of tumor-infiltrating CD8 T cells in NSCLC patients (*n* = 32) revealed increased expression of immune inhibitory molecules PD-1, TIM-3, CTLA-4, LAG-3, and BTLA correlating with T cell dysfunction and tumor progression (Thommen et al. 2015), and expression of these immune inhibitory checkpoints may exert an immunosuppressive state despite anti-PD-1 treatment. Profiling of peripheral blood from melanoma patients before and after treatment with anti-PD-1 identified an exhausted CD8 T cell phenotype, which can be reinvigorated with treatment. Interestingly, resistance to anti-PD-1 was not due to a failure in reinvigoration, but rather the magnitude of the reinvigoration in relation to tumor burden (Huang et al. 2017). Exhausted CD8 T cells show sustained levels of PD-1 and a distinct epigenetic landscape compared to other subsets of effector and memory CD8 T cells (Sen et al. 2016); difference in the epigenetic modulation of this exhausted T cell phenotype affects reinvigoration with anti-PD-1 therapy (Pauken et al. 2016), and their ability to be reprogrammed to avoid exhaustion and dysfunction (Philip et al. 2017).

### Presence of immunosuppressive cells

Immune cells such as the T regulatory and Th2 T cells, myeloid-derived suppressor cells and M2 polarized and tumor-associated macrophages secrete various anti-inflammatory and immune suppressive factors to dampen T cell-mediated responses. Hence, identification of these cells in the tumor microenvironment using various immune profiling techniques has shed light on why some patients fail immune checkpoint therapy. Indeed, depletion of T regulatory cells has been shown to improve anti-tumor immune response (Viehl et al. 2006), while presence of myeloid-derived suppressor cells correlated with decreased anti-CTLA-4 response (Meyer et al. 2014). Intra-tumoral macrophages were identified in tumors resistant to immune checkpoint blockade in a mouse breast carcinoma model, and targeting of these cells using a selective PI3K inhibitor increased anti-tumor T cell activity (De Henau et al. 2016). Tumor-associated macrophages were also shown to scavenge anti-PD-1, and the removal of PD-1 antibodies from CD8 T cells may impede treatment efficacy (Arlauckas et al. 2017). Targeting of tumor-associated macrophages with anti-CSF-1R reduced their infiltration, and when used in combination with anti-PD-1 or anti-CTLA-4, enhanced tumor regression (Zhu et al. 2014).

## Conclusions

The profile and distribution of immune cells play essential roles in determining response to immune checkpoint blockade. Characterization of the immune microenvironment has shown enormous value in identifying prognostic and predictive immune-associated biomarkers that can be implemented in the clinic for patient care. Moreover, immune profiling of resistant tumor biopsies has elucidated the intricate biological processes contributing to failure to immune checkpoint inhibitors, and uncovering these resistance mechanisms will reveal strategies to improve the efficacy of these therapies.
